# Friedenthal Milk

**Published:** 1915-10

**Authors:** 


**Friedenthal Milk.** E. A. Risenfeld of N. Y., Arch. of Paed., Aug., gives Friedenthal's formula: Skimmed milk, 330 c.c., Water, 660 c.c., Lactose 68.9 G., Molkerei salts, 1.89 G., Fat, in cream, 4.5 per cent. Molkerei salts consist of Potassium chlorid 2, Potassium phosphate 1, Potassium biphosphate 1. In Riesenfeld's experiments, a good grade of milk was used, neither pasteurized nor boiled. He substituted saccharose or dextri-maltose for lactose, with better result. Schloss is quoted also as having poor results with lactose.

